# Identification of a species-specific aminotransferase in *Pediococcus acidilactici* capable of forming α-aminobutyrate

**DOI:** 10.1186/s13568-020-01034-2

**Published:** 2020-05-29

**Authors:** Alexander Wenger, Remo S. Schmidt, Reto Portmann, Alexandra Roetschi, Elisabeth Eugster, Laure Weisskopf, Stefan Irmler

**Affiliations:** 1 Agroscope, Schwarzenburgstrasse 161, 3003 Bern, Switzerland; 2 Department of Biology, University of Fribourg, Rue Albert-Gockel 3, 1700 Fribourg, Switzerland; 3 Bern University of Applied Sciences, School of Agricultural, Forest, and Food Sciences HAFL, Länggasse 85, 3052 Zollikofen, Switzerland

**Keywords:** *Pediococcus acidilactici*, α-Aminobutyrate, α-Ketobutyrate, Aminotransferase

## Abstract

During cheese ripening, the bacterial strain *Pediococcus acidilactici* FAM18098 produces the non-proteinogenic amino acid, α-aminobutyrate (AABA). The metabolic processes that lead to the biosynthesis of this compound are unknown. In this study, 10 *P. acidilactici*, including FAM18098 and nine *Pediococcus pentosaceus* strains, were screened for their ability to produce AABA. All *P. acidilactici* strains produced AABA, whereas the *P. pentosaceus* strains did not. The genomes of the pediococcal strains were sequenced and searched for genes encoding aminotransferases to test the hypothesis that AABA could result from the transamination of α-ketobutyrate. A GenBank and KEGG database search revealed the presence of a species-specific aminotransferase in *P. acidilactici*. The gene was cloned and its gene product was produced as a His-tagged fusion protein in *Escherichia coli* to determine the substrate specificity of this enzyme. The purified recombinant protein showed aminotransferase activity at pH 5.5. It catalyzed the transfer of the amino group from leucine, methionine, AABA, alanine, cysteine, and phenylalanine to the amino group acceptor α-ketoglutarate. Αlpha-ketobutyrate could replace α-ketoglutarate as an amino group acceptor. In this case, AABA was produced at significantly higher levels than glutamate. The results of this study show that *P. acidilactici* possesses a novel aminotransferase that might play a role in cheese biochemistry and has the potential to be used in biotechnological processes for the production of AABA.

## Key points

- *Pediococcus acidilactici* possesses a species-specific aminotransferase.
- The aminotransferase catalyzes the reversible transfer of an amino group to α-keto butyrate to form α-aminobutyrate.
- The aminotransferase uses a variety of amino acids as amino group donor.
- The aminotransferase is active at pH 5.5, a pH encountered in cheese.

## Introduction

During cheese ripening, proteolysis of the caseins occurs. In a series of degradation steps, the proteins are broken down into amino acids. Thereby, lactic acid bacteria (LAB) play an important role as they use the free amino acids for energy production, regulation of the internal pH, regeneration of co-substrates, and protein biosynthesis (Ardö 2006).

Recently, it was shown that *Pediococcus acidilactici* FAM18098 catabolized serine and threonine and formed α-aminobutyrate (AABA) and alanine in vitro and in cheese (Irmler et al. 2013; Eugster et al. 2019). Especially, the formation of the non-proteinogenic amino acid, AABA, is quite unusual, and the enzymes involved in the anabolism of this substance are unknown. It was shown that the compound could be synthesized in metabolically engineered bacteria by using enzymes that either transaminate or reduce α-ketobutyrate (AKB) (Fotheringham et al. 1999; Zhang et al. 2010).

However, AABA formation also occurs naturally, for example in *P. acidilactici*. It has been suggested that in *P. acidilactici*, threonine is the precursor (Irmler et al. 2013; Eugster et al. 2019). Threonine could be deaminated to AKB, which is then aminated or transaminated to AABA. Likewise, serine could be converted via pyruvate to alanine. These reaction steps are similar to the ones used in the engineered bacteria, where either a transaminase or a dehydrogenase catalyzes the final step of AABA synthesis (Fotheringham et al. 1999; Zhang et al. 2010).

*Pediococcus acidilactici* belongs to the LAB and ferments sugar mainly to lactate (Holzapfel et al. 2006). The aminotransferase activity has been studied in cells or cell-free extract of other LAB such as *Lactococcus lactis* (Engels et al. 2000), *Lactobacillus paracasei* (Williams et al. 2002; Thage et al. 2004a), *Lactobacillus helveticus* (Klein et al. 2001; Thage et al. 2004a), and *Lactobacillus plantarum* (Nierop Groot and de Bont 1998). Furthermore, data of purified enyzmes from *L. lactis* and *L. paracasei* are available (Yvon et al. 1997, 2000; Thage et al. 2004b). All these studies have in common that α-ketoglutarate (AKG) is the preferred amino group acceptor.

However, as can be judged from the KEGG PATHWAY database (http://www.genome.jp/kegg/pathway.html), *P. acidilactici* is not able to synthesize AKG. That means that AKG can neither be synthesized by the citrate cycle nor from glutamate since the genes encoding aconitase (EC 4.2.1.3), isocitrate dehydrogenase (EC 1.1.1.41), and glutamate dehydrogenase (EC 1.4.1.2) are not present. Therefore, it is very likely that aminotransferases present in *P. acidilactici* utilize other amino group acceptors.

In this study, nine *P. acidilactici* and nine *P. pentosaceus* strains isolated from dairy products were screened for their ability to produce AABA. *P. acidilactici* FAM18098 served as a control strain. Furthermore, the genomes were sequenced and searched for genes encoding amino acid aminotransferases. Finally, the gene of a *P. acidilactici*-specific aminotransferase was cloned, and the substrate specificity of this enzyme was determined.

## Materials and methods

### Bacterial strains, media, and growth conditions

The bacterial strains used in this study are listed in Table 1. Pediococci were cultivated in MRS broth (de Man et al. 1960) at 30 °C. The capability to produce AABA was assessed by the following procedure: the bacteria were grown at 30 °C for 3 d in a basal broth (pH 7.0 ± 0.2) that consisted of di-potassium hydrogen phosphate (9 g L^−1^), yeast extract (5 g L^−1^), casein hydrolysate (2 g L^−1^), magnesium sulfate (0.2 g L^−1^), manganese chloride (0.2 g L^−1^), d-galactose (2 g L^−1^), 5 mM l-serine, and 5 mM l-threonine.

**Table 1:** Strains and plasmids used in this study

Strain	GenBank acc. no., genotype, relevant properties	Source
*E. coli*		
TOP10	F- *mcrA* Δ(*mrr*-*hsd*RMS-*mcr*BC) Φ80*lac*ZΔM15 Δ *lac*X74 *rec*A1 *ara*D139 Δ(*araleu*)7697 *gal*U *gal*K *rps*L (StrR) *end*A1 *nup*G	Invitrogen
BL21(DE3)	F- *ompThsdS*_B_(r_B_^−^ m_B_^−^) *galdcm* (DE3)	Invitrogen
BL21/aat	*E. coli* BL21(DE3) harboring pEXP5-CT/*aat*	This study
*P. acidilactici*		
FAM 18098	GCA_009808095.1	Agroscope culture collection
FAM 13473	GCA_009809395.1	Agroscope culture collection
FAM 13881	GCA_009809715.1	Agroscope culture collection
FAM 17411	GCA_009809065.1	Agroscope culture collection
FAM 17418	GCA_009809885.1	Agroscope culture collection
FAM 17422	GCA_009808325.1	Agroscope culture collection
DSM 20284^T^	GCA_000146325.1	German collection of microorganisms and cell cultures
FAM 18987	GCA_009809575.1	Agroscope culture collection
FAM 19460	GCA_009809605.1	Agroscope culture collection
FAM 20559	GCA_009808215.1	Agroscope culture collection
*P. pentosaceus*		
FAM 13073	GCA_009809665.1	Agroscope Culture Collection
FAM 17622	GCA_009809195.1	Agroscope culture collection
FAM 18813	GCA_009808845.1	Agroscope culture collection
FAM 19080	GCA_009809425.1	Agroscope culture collection
FAM 19132	GCA_009809595.1	Agroscope culture collection
FAM 19144	GCA_005864405.1	Agroscope culture collection
FAM 19169	GCA_009808085.1	Agroscope culture collection
FAM 20650	GCA_009808815.1	Agroscope culture collection
DSM 20336^T^	GCA_001437285.1	German collection of microorganisms and cell cultures
Plasmids		
pEXP5-CT/TOPO	*E. coli* cloning vector, Amp^r^	Invitrogen
pEXP5-CT/*aat*	Plasmid expressing the aminotransferase gene *aat* from FAM18098, Amp^r^	This study

^T^: type strain

*Escherichia coli* strains were grown in LB broth (Sambrook et al. 1989) supplemented with ampicillin (50 µg mL^−1^) if necessary, with shaking (220 rpm) at 37 °C.

### Determination of free amino acids

High-performance liquid chromatography (HPLC) was used to determine the free amino acids in culture supernatants and enzyme assays. Therefore, 100 µL of the sample was mixed with 1 mL of 100 mM of HCl, 0.5 mL of 0.5 µM of l-norvalin, 0.5 mL of 0.5 µM of piperidin, and 0.5 mL of 10% (w/v) of trichloroacetic acid and incubated for 30 min at 5 °C. After centrifugation, the amino acids present in the supernatant were determined using HPLC as described previously (Wenzel et al. 2018).

### Genome sequencing and bioinformatic analysis

Genomic DNA was extracted as described in Berthoud et al. (2017), and DNA concentration was determined using the Qubit dsDNA BR Assay Kit (Thermo Fisher Scientific, Reinach, Switzerland). The genomes of *P. acidilactici* FAM18098 and *P. pentosaceus* FAM19132 were sequenced using PacBio sequencing. The genomes of the other nine *P. acidilactici* strains and eight *P. pentosaceus* strains were determined using Illumina technology. The protocol for genome sequencing and assembly was performed as described previously (Wuethrich et al. 2017).

The annotated genome sequence of strain FAM18098 (Genbank acc. no. GCA_009808095.1) was retrieved from the FTP directory for GenBank assemblies (https://ftp.ncbi.nlm.nih.gov/genomes/all/GCA/009/808/095/GCA_009808095.1_ASM980809v1/) and searched for genes encoding aminotransferases. Similarity searches with genes of interest were then performed by running BLAST searches (Altschul et al. 1990) against the GenBank (https://blast.ncbi.nlm.nih.gov/Blast.cgi) and the KEGG GENES database (https://www.genome.jp/tools/blast/). Furthermore, BLAST searches were carried out against a custom BLAST database containing the newly generated genome assemblies of the *Pediococcus* strains whose accession numbers are listed in Table 1. Clustal Omega was used for multiple sequence alignments (Sievers et al. 2011).

### Cloning and heterologous gene expression

The primers aat_Fw (5′-ATGAGTGATAAAGTTAACGC-3′) and aat_Rv (5′-TGCTCGTTTAGCTTTGAGG-3′) were designed to amplify the complete aminotransferase-coding gene presented by the locus GBO44_RS03485. The primer aat_Rv did not contain the stop codon for cloning reasons. The amplification reactions were performed using the AmpliTaq Gold DNA Polymerase (Thermo Fisher Scientific) and applying the manufacturer’s protocol. Five nanograms of genomic DNA were used in 25 µL reaction volume. The amplification occurred in a thermal cycler using the following program: 10 min at 95 °C; 30 cycles of 15 s at 95 °C, 30 s at 45 °C, and 1 min at 72 °C; 5 min at 72 °C. The reaction products were analyzed using agarose gel electrophoresis.

The DNA fragment obtained from strain FAM18098 was cloned into the pEXP5-CT/TOPO expression vector (Thermo Fisher Scientific) applying the manufacturer’s protocol. The vector contains a nucleotide sequence encoding a poly-histidine tag that is added to the 3′-end of the DNA fragment. The recombinant vector was first transformed into *E. coli* TOP10. The plasmids extracted from transformants were verified using Sanger sequencing at Microsynth (Balgach, Switzerland). A plasmid containing the DNA fragment in the proper orientation was named pEXP5-CT/*aat* and transformed into *E. coli* One Shot BL21(DE3) (Thermo Fisher Scientific) for protein production.

### Production and purification of recombinant protein

An overnight culture of *E. coli* BL21(DE3) harboring the plasmid pEXP5-CT/*aat* was used to inoculate 200 mL of LB broth supplemented with ampicillin. The culture was incubated with shaking (220 rpm) at 37 °C. When the culture reached an optical density at 600 nm of 0.4, 0.5 mM of isopropyl-beta-d-1-thiogalactoside (IPTG) was added to induce gene expression. The culture was then incubated with shaking at 30 °C for 4 h. The bacteria were harvested by centrifugation (3000×*g*, 10 min, RT), washed with 20 mM of sodium phosphate (pH 7.4), and frozen at − 20 °C.

The His-tagged recombinant protein was purified using Protino Ni-TED 1000 Packed columns (Machery-Nagel, Oensingen, Switzerland) according to the manufacturer’s instructions. The cells were disrupted in 1 mL of LEW buffer provided by the Protino Ni-TED columns kit to which approximately 0.4 g of glass beads (212–300 µm in diameter) and 250 U of Benzonase Nuclease (Grogg Chemie AG, Stettlen-Deisswil, Switzerland) were added using an Omni Bead Ruptor (6 m^−1^, 45 s). The slurry was cleared by centrifugation and the supernatant was used for protein purification. The fraction containing the His-tagged protein was immediately changed to 20 mM of sodium phosphate (pH 7.4) using illustra NAP columns (VWR International GmbH, Dietikon, Switzerland). The protein concentration was determined using the Qubit Protein Assay kit (Thermo Fisher Scientific, Waltham, USA). The purity of the purified protein was evaluated using denaturating gel electrophoresis. The proteins within the gel were visualized using QuickBlue Protein Stain (LuBioScience GmbH, Lucerne, Switzerland).

### Enzymatic assays

The ability to transfer the amino group from an amino acid to AKG was studied using a coupled-enzyme assay. In the first reaction, the purified His-tagged protein was incubated with either l-aspartate, l-methionine, l-cysteine, l-leucine, l-isoleucine, l-valine, l-phenylalanine, l-serine, l-threonine, l-alanine, or AABA as amino group donor and AKG as amino group acceptor. In the second reaction, the amount of glutamate, which resulted from the transamination of AKG, was measured using glutamate dehydrogenases. The first reaction (200 µL) consisted of 50 mM of potassium phosphate (pH 5.5), 5 mM of amino acid, 10 mM of AKG, 50 µM of pyridoxal-5-phosphate, and enzyme (8 µg). After incubation at 37 °C for an hour, 20 µL of the sample was used for the second reaction. The sample was added to 180 µL of reaction buffer (50 mM of potassium phosphate, 50 mM of triethanolamine, 1% [w/v] of Triton X-100, [pH 9.0]), 0.12 mM of iodonitrotetrazolium, 1.73 mM of NAD^+^, NAD-dependent glutamate dehydrogenase (1.8 U), and diaphorase (17.6 mU). After an incubation at 37 °C for 30 min, the absorption at 492 nm was determined. Control reactions did not include the His-tagged recombinant protein. The absorption from the control reactions was subtracted from the corresponding reactions containing the His-tagged protein. For the calculation of relative activities, the assay with leucine/AKG was set at 100%. Sodium chloride was added in steps of 1% (w/v) to the first reaction to study the influence of salt. 50 mM of potassium phosphate buffer with pH 7.4 and pH 9.1 were used in the first reaction instead of pH 5.5 to measure the influence of pH.

To study if α-keto acids other than AKG act as substrates, α-ketobutyrate (AKB), pyruvate, phenylpyruvate (PPA), α-keto-γ-(methylthio)butyrate (KMTB), or α-ketoisocaproate (KIC) were used instead of AKG in the aforementioned first reaction. After incubation at 37 °C for 1 h, the presence of amino acids was determined using HPLC as described above. The activities were compared by dividing the amount of the amino acid formed by the amount of the total amino acids present in the sample.

Statistical analysis was carried out in the statistical program R (version 3.6.2, available at https://www.r-project.org/). Significance values were calculated by computing Tukey’s honest significant differences (HSD) after fitting an analysis of variance model to the data.

## Results

### *Pediococcus acidilactici* produces the non-proteinogenic amino acids, AABA and ornithine

**Fig. 1:**
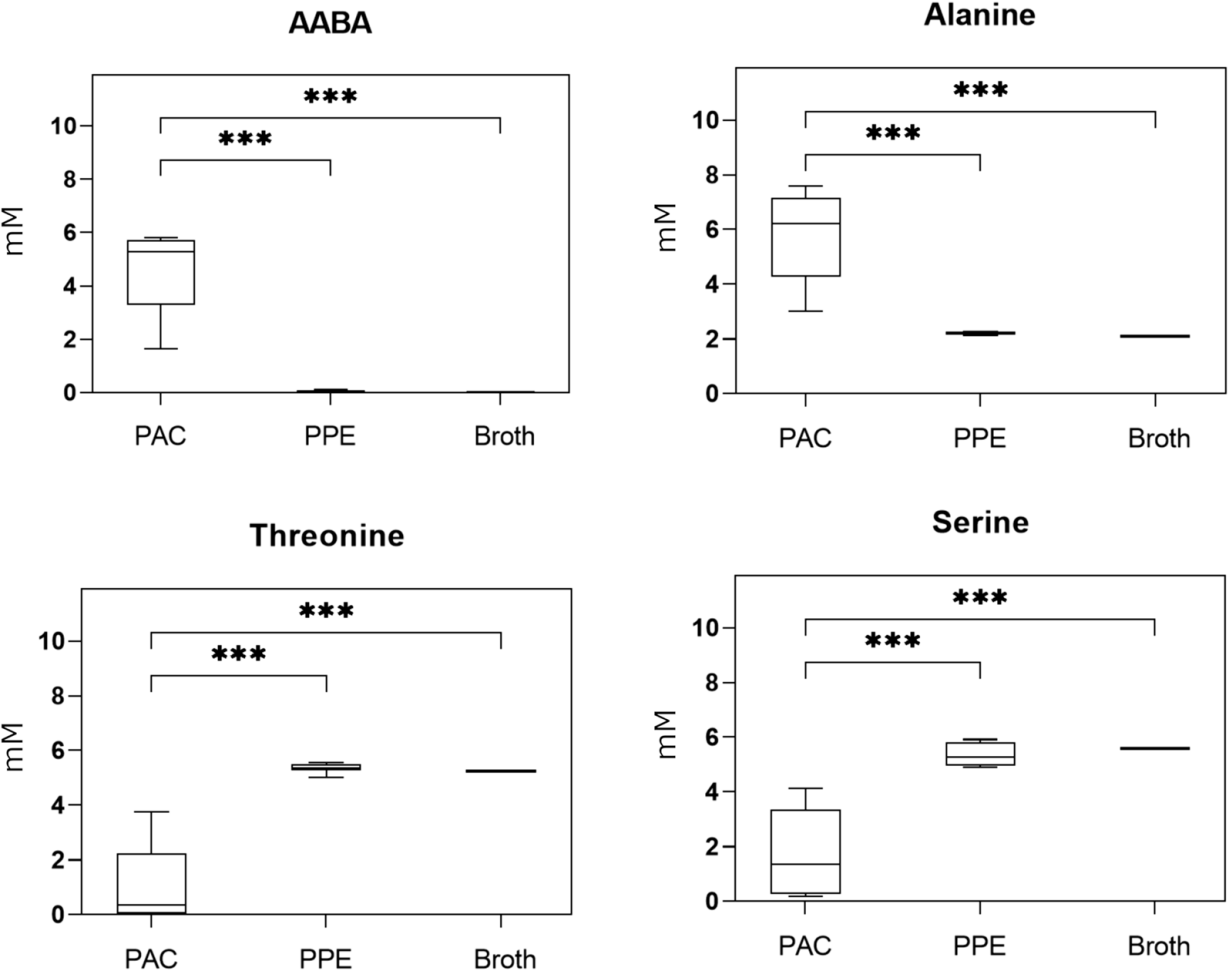
Boxplot of AABA, threonine, alanine, and serine concentrations determined in the culture supernatants of *Pediococcus acidilactici* (PAC) and of *Pediococcus pentosaceus* (PPE) and in uninoculated broth. Data include the mean of two independently performed fermentations. Asterisks indicate significant differences (***P < 0.001)

*Pediococcus acidilactici* FAM18098 and nine other *P. acidilactici* strains (Table 1) were incubated in a broth in which AABA formation can be detected to test if AABA formation was strain- or species-specific. Additionally, nine strains of *P. pentosaceus* were included in this analysis. The analysis of the free amino acids after 3 days of incubation showed that AABA and alanine was formed in all of the samples with *P. acidilactici* (Fig. 1). Concomitantly, serine and threonine were degraded. In addition, the concentration of phenylalanine was on average 0.2 mM lower than in the non-inoculated medium (data now shown). The strains FAM13881 and FAM18987 produced less alanine and AABA in comparison to the other *P. acidilactici* strains. Accordingly, the levels for serine and threonine were higher in both strains compared to the other *P. acidilactici* strains. These amino acid changes were not observed in the samples with *P. pentosaceus* (Fig. 1).

Furthermore, the culture supernatant of all pediococci contained ornithine whereas arginine was not detected any more (Additional file 1: Table S1).

### In silico analysis of aminotransferases

**Fig. 2:**
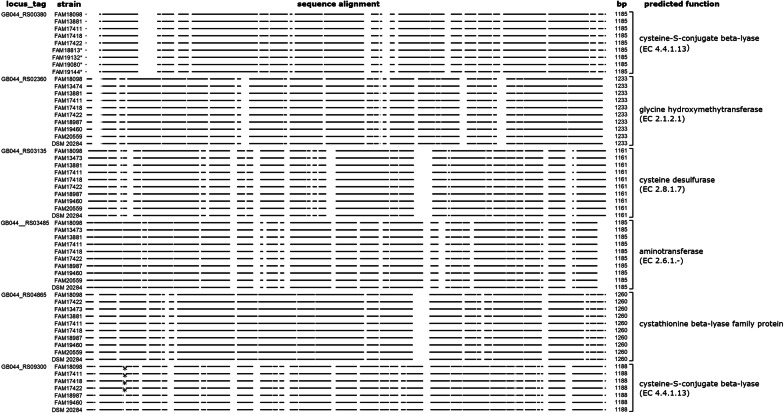
Bioinformatic analysis of the aminotransferases found in *P. acidilactici*. The gene sequences of six locus_tags that encode aminotransferases in *P. acidilactici* FAM18098 were used to find orthologs in the genomes of the study strains. Orthologs of GB044_RS00380 were also found in the genomes of the *P. pentosaceus* strains, which are indicated by an asterisk. The horizontal lines represent aligning nucleotide sequences. The predicted functions are based on sequence similarity searches against the KEGG GENES database

The genome data of strain FAM18098 was searched for genes encoding aminotransferases to follow this hypothesis. Six genes represented by GBO44_RS00380, GBO44_RS02360, GBO44_RS03135, GBO44_RS03485, GBO44_RS04865, and GBO44_RS09300 were annotated to encode amino acid aminotransferases (Fig. 2). BLAST searches were performed to obtain more clarity about the function of these aminotransferases. First, it was found that the two genes GBO44_RS00380 and GBO44_RS09300 were not present in all 10 *P. acidilactici* strains. In addition, orthologs of GBO44_RS00380 were found in four *P. pentosaceus* genomes. Second, one of the remaining four aminotransferases, GBO44_RS02360, was predicted to be a glycine hydroxymethyltransferase, an enzyme that catalyzes the interconversion of glycine and 5,10-methylenetetrahydrofolate to serine and tetrahydrofolate. Third, the two aminotransferases encoded by GBO44_RS03135 and GBO44_RS04865 were predicted to be involved in the metabolism of sulfur-containing amino acids. Finally, no function could be predicted from the sequence alignments of the last aminotransferase, GBO44_RS04865. The nearest protein homolog in the phylogenetically closely related *P. pentosaceus* showed only 46% identity (data not shown), confirming that this enzyme was specific to *P. acidilactici*. The gene was cloned and heterologously expressed in *E. coli* to obtain enzymatic data on this aminotransferase.

### Enzymatic properties of the recombinant aminotransferase

The primers were designed based on the nucleotide sequence GBO44_RS04865, which is named *aat* in the following. When the primers were tested on genomic DNA of 10 *P. acidilactici* strains, a DNA fragment with a size of approximately 1200 bp was obtained from all *P. acidilactici* strains except for FAM18987 (data not shown). An alignment of the *aat* gene sequences showed that the binding site for the primer aat_Rv displayed multiple mismatches in strain FAM18987 (Additional file 2: Figure S1), explaining why the gene could not be amplified from this strain.

**Fig. 3:**
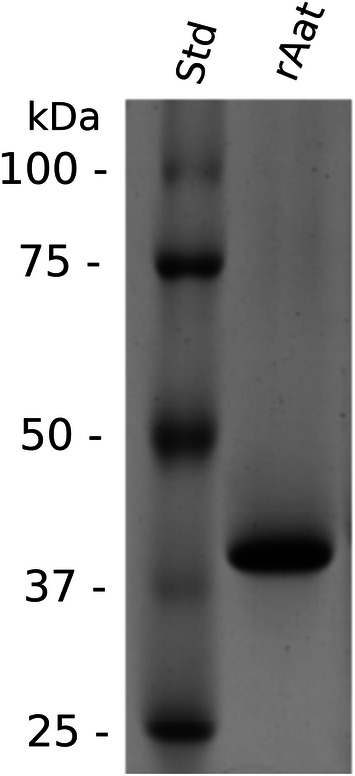
Gel electrophoretic analysis of the purified, His-tagged aminotransferase. Lanes: 1, protein standard; 2, purified, recombinant aminotransferase Aat (expected weight 44.9 kDa)

**Fig. 4:**
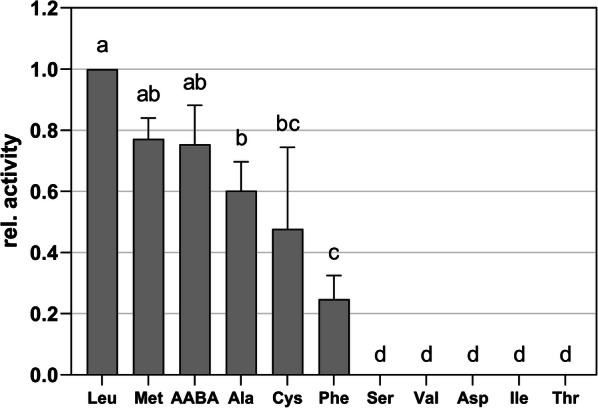
Relative activity of the recombinant aminotransferase with leucine (Leu), methionine (Met), α-aminobutyrate (AABA), alanine (Ala), cysteine (Cys), phenylalanine (Phe), serine (Ser), valine (Val), aspartate (Asp), isoleucine (Ile), and threonine (Thr), as amino group donor and α-ketoglutarate as amino group acceptor at pH 5.5. The highest activity was found with leucine, which was set at 100% for the calculation of the relative activities. The column height represents the mean (± SD) of triplicate measurements. The letters above the columns indicate significant differences between amino group donors (P < 0.05)

The *aat* gene of FAM18098 was cloned, and the encoded gene product was produced as a His-tagged fusion protein in *E. coli.* The protein could be purified to apparent homogeneity using nickel affinity chromatography (Fig. 3). First, the purified protein was tested for aminotransferase activity using AKG as amino group acceptor. It was found that the enzyme transferred the amino group from leucine, methionine, AABA, alanine, cysteine, and phenylalanine to AKG (Fig. 4).

When the influence of pH and salt concentration was studied, activity was only found at pH 5.5 but not at pH 7.4 or pH 9.1. An increase of the sodium chloride concentration to 4% (w/v) led to a reduction in activity of approximately 22% (data not shown).

**Fig. 5:**
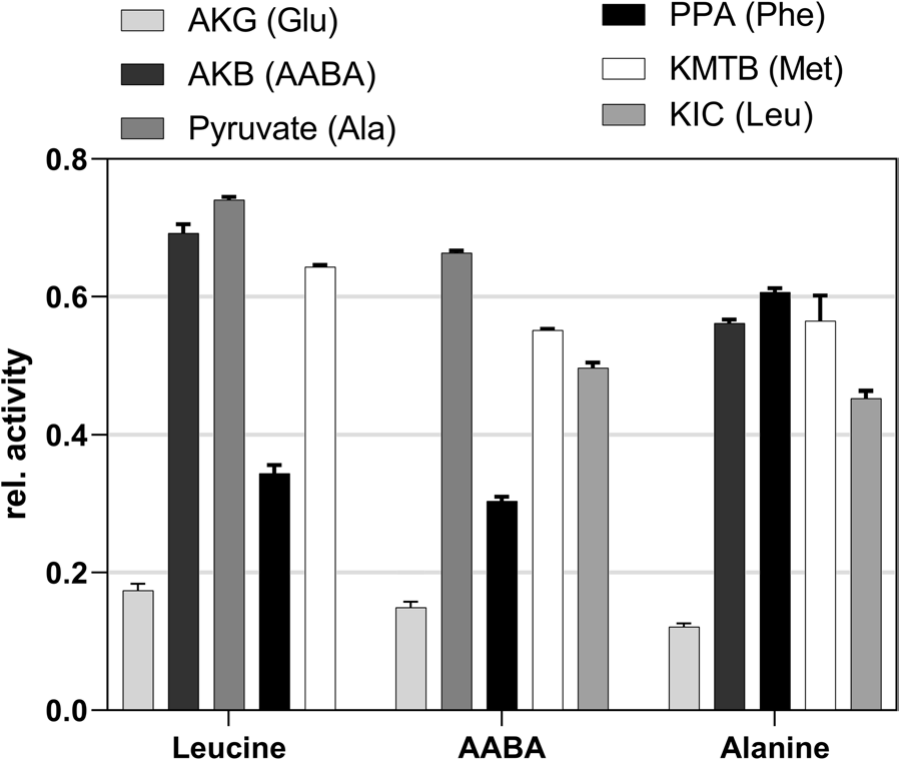
Utilization of the amino group acceptors α-ketoglutarate (AKG), α-ketobutyrate (AKB), pyruvate, phenylpyruvate (PPA), α-keto-γ-methylthiobutyrate (KMTB), and α-ketoisocaproate (KIC). The relative activity represents the ratio of the amount of the amino acid formed (shown in the legend in the parenthesis) and the amount of total free amino acids. The column height represents the mean (± SD) of duplicate measurements

Then, various α-keto acids were used as amino group acceptors. As mentioned before, no evidence for the biosynthesis of AKG in *P. acidilactici* can be found based on annotated genome data. This lack of evidence indicates that the aminotransferases of *P. acidilactici* may utilize other amino group acceptors. The amino group acceptors AKG, AKB, pyruvate, PPA, KMTB, and KIC were compared using leucine, AABA, and alanine as amino group donors. It was found that AKB, pyruvate, KMTB, KIC, and PPA significantly preferred amino group acceptors to AKG (Fig. 5).

## Discussion

*Pediococcus acidilactici* FAM18098 was shown previously to produce AABA in broth and cheese (Irmler et al. 2013; Eugster et al. 2019). The present study shows that this phenotype is widely distributed within the species, since other *P. acidilactici* strains also produced the compound and, to our knowledge, has not been reported for other LAB species. Additionally, all *P. acidilactici* strains produced alanine and degraded serine and threonine, suggesting these two latter amino acids are the precursors for alanine and AABA formation. This hypothesis is supported by the observation that the two strains FAM13881 and FAM18987 that produced less alanine and AABA in comparison to the other *P. acidilactici* strains, concomitantly utilized less serine and threonine.

Together with the degradation of serine and threonine, a significant decrease in the concentration of phenylalanine was observed in the samples with *P. acidilactici*. However, since the affected concentrations of phenylalanine were in the micromolar range and since this decrease was not observed in cheese (Eugster et al. 2019), it can be assumed that the degradation of phenylalanine, if at all, contributes only a minimal amount to the biosynthesis of alanine or AABA.

All inoculated samples also contained ornithine after 3 days of incubation. Ornithine results from the arginine deiminase pathway, which involves the sequential action of arginine deiminase and ornithine transcarbamylase that convert arginine via citrulline to ornithine (Liu et al. 1995). Consequently, arginine was not detected anymore at the end of incubation. This finding is in agreement with the observation that the hydrolysis of arginine is a common phenotypic characteristic of *P. acidilactici* and *P. pentosaceus* within the genus *Pediococcus* (Holzapfel et al. 2006).

AABA formation in *E. coli* was achieved by introducing plasmids harboring a threonine deaminase and an aminotransferase into the cells (Fotheringham et al. 1999). Through the concerted action of both enzymes, threonine was converted via AKB to AABA. Analogously, it can be hypothesized that *P. acidilactici* synthesizes AABA from threonine.

When the genomes of the study strains were searched for genes encoding these two enzymes, a gene encoding a bifunctional threonine ammonia-lyase/l-serine ammonia-lyase (data not shown) and genes encoding aminotransferases were found in the genome sequences of *P. acidilactici*. Amongst the aminotransferases, one gene (GB044_RS04865) was present in all *P. acidilactici* strains, of which the function could not predicted using database searches. The present study focused on this enzyme.

The purified, recombinant aminotransferase was active under conditions encountered in a cheese environment (low pH, elevated salt concentrations). Furthermore, the activity of the aminotransferase was found to be significantly higher with pyruvate, AKB, and KMTB than with AKG using leucine as amino group donor (Fig. 5). This is in line with the previously mentioned fact that a pathway for AKG biosynthesis does not exist in *P. acidilactici.* It can be speculated that amino acid-catabolizing enzymes such as the bifunctional serine/threonine deaminase or cystathionine/methionine lyases provide the amino group acceptors necessary for the aminotransferase activity in *P. acidilactici.*

Data about the substrate specificity of LAB aminotransferases is available from three other species. The branch-chained aminotransferase BcaT from *L. lactis* (Yvon et al. 2000) utilized mainly the amino acids, isoleucine, leucine, valine, and methionine, and clearly preferred AKG as amino group acceptor, used KIC to a lesser extent, and displayed only low activity with pyruvate. A branched-chain aminotransferase with similar activities has been purified from *L. paracasei* (Thage et al. 2004b). This enzyme degraded isoleucine, leucine, and valine at similar rates. In addition, the enzyme used AKG at high rates and only low activities were observed with pyruvate and AKB as amino group acceptors. The AraT aminotransferase from *L. lactis* (Yvon et al. 1997*)* showed high activity towards leucine and phenylalanine as amino group donor, and AKG and KIC as amino group acceptors. Furthermore, AraT exhibited no activity with valine, isoleucine, cysteine, and alanine. Unfortunately, neither AABA nor AKB were tested as substrates for AraT in this study.

In contrast to the aforementioned branched-chain aminotransferases, the Aat from *P. acidilactici* showed no activity with valine and isoleucine and significantly preferred AKB, pyruvate, KMTB, KIC, and PPA as amino group acceptor. In contrast to AraT, the pediococcal aminotransferase newly described in this study showed activity towards cysteine and alanine (Fig. 4). Therefore, this aminotransferase apparently represents a new aminotransferase class within the LAB with promising new properties.

With regard to the fermentative production of AABA using *P. acidilactici* it has to be considered that the structure and chemical properties of alanine are similar to the ones of AABA. This may introduce difficulties in the separation and purification of AABA. Therefore, *P. acidilactici* probably cannot be used directly for industrial processes as long as serine is present in the starting material for fermentation.

However, the bacterium obviously possesses genes with biosynthetic activities that can be used for metabolic engineering purposes. Based on the capability of the Aat aminotransferase to use alanine as amino group donor for AABA synthesis, the following hypothetical pathway that involves the action of three enzymes could be constructed: the bifunctional threonine/serine deaminase of *P. acidilactici* would degrade threonine and serine to AKB and pyruvate, respectively. Pyruvate could then be converted to alanine in a reductive amination reaction involving an alanine dehydrogenase. Interestingly, two genes encoding alanine dehydrogenases are present in the genomes of *P. acidilactici* (data not shown), which could be used for this approach. Finally, the *P. acidilactici* Aat could use AKB and alanine to produce AABA and pyruvate, respectively. The latter reaction product would be recycled to alanine by the alanine dehydrogenase. In theory, this could shift the equilibrium of the transamination reaction towards the reaction product AABA. To test this hypothetical metabolic pathway, it is planned to clone the corresponding genes from *P. acidilactici* and couple the biosynthetic activities by co-expression of the genes in *E. coli.*

## Supplementary information

**Additional file 1: Figure S1.** Sequence alignment of the aat gene from different *Pediococcus acidilactici* strains. The two primers aat_Fw and aat_Rv were used in the PCR reactions.

**Additional file 2: Table S1.** Concentrations of the free amino acids (mmol/L) in broth fermented with different pediococcal strains. The values of two independently performed experiments are shown. Basal broth stands for the non-inoculated medium. (AABA: α-aminobutyrate, GABA: γ-aminobutyrate, FAA: free amino acids).

## Data Availability

Corresponding author could provide all the experimental data on valid request.
